# Using informative prior based on expert opinion in Bayesian estimation of the transition probability matrix in Markov modelling—an example from the cost-effectiveness analysis of the treatment of patients with predominantly negative symptoms of schizophrenia with cariprazine

**DOI:** 10.1186/s12962-020-00224-w

**Published:** 2020-08-27

**Authors:** Zoltán Vokó, István Bitter, Beatrix Mersich, János Réthelyi, Anett Molnár, János G. Pitter, Árpád Götze, Margit Horváth, Kristóf Kóczián, Laura Fonticoli, Filippo Lelli, Bertalan Németh

**Affiliations:** 1 Center for Health Technology Assessment, Semmelweis University, Üllői út 25, 1091 Budapest, Hungary; 2 Syreon Research Institute, Mexikói út 65/A, 1142 Budapest, Hungary; 3 Department of Psychiatry and Psychotherapy, Semmelweis University, Balassa utca 6, 1083 Budapest, Hungary; 4 Richter Gedeon Plc, Gyömrői út 19-21, 1103 Budapest, Hungary; 5 Recordati S.P.A, Via Matteo Civitali 1, 20148 Milano, MI Italy

**Keywords:** Bayesian statistics, Transition probabilities, Markov model, Schizophrenia

## Abstract

**Background:**

When patient health state transition evidence is missing from clinical literature, analysts are inclined to make simple assumptions to complete the transition matrices within a health economic model. Our aim was to provide a solution for estimating transition matrices by the Bayesian statistical method within a health economic model when empirical evidence is lacking.

**Methods:**

We used a previously published cost-effectiveness analysis of the use of cariprazine compared to that of risperidone in patients with predominantly negative symptoms of schizophrenia. We generated the treatment-specific state transition probability matrices in three different ways: (1) based only on the observed clinical trial data; (2) based on Bayesian estimation where prior transition probabilities came from experts’ opinions; and (3) based on Bayesian estimation with vague prior transition probabilities (i.e., assigning equal prior probabilities to the missing transitions from one state to the others). For the second approach, we elicited Dirichlet prior distributions by three clinical experts. We compared the transition probability matrices and the incremental quality-adjusted life years (QALYs) across the three approaches.

**Results:**

The estimates of the prior transition probabilities from the experts were feasible to obtain and showed considerable consistency with the clinical trial data. As expected, the estimated health benefit of the treatments was different when only the clinical trial data were considered (QALY difference 0.0260), its combination with the experts’ beliefs were used in the economic model (QALY difference 0.0253), and when vague prior distributions were used (QALY difference 0.0243).

**Conclusions:**

Imputing zeros to missing transition probabilities in Markov models might be untenable from the clinical perspective and may result in inappropriate estimates. Bayesian statistics provides an appropriate framework for imputing missing values without making overly simple assumptions. Informative priors based on expert opinions might be more appropriate than vague priors.

## `Background

One of the most widely used methods of cost-effectiveness modelling in healthcare is Markov modelling [1]. Markov models are state transition models in which the life course of a cohort of subjects or a series of individuals are modelled by placing patients into discrete and mutually exclusive health states. Utilities and costs are assigned to each state and time period so that expected utilities and costs can be estimated [2].

In these models, the health state transition probabilities (from one time period to the next) are usually estimated from empirical studies (clinical trials, observational epidemiological studies, or a meta-analysis of these studies). Sometimes data on some transition probabilities might be lacking entirely, simply because those transitions were not observed or reported in the clinical studies. An easy modelling solution for unobserved transitions is to assume that they never happen and assign a zero value to these transition probabilities. Nevertheless, this zero-value assignment approach may not be reasonable from the clinical perspective since such health state transitions occur in clinical practice within large samples or long time periods.

A natural way to address this missing evidence issue comes from Bayesian statistics, which allows us to combine prior beliefs and evidence formally and quantitatively to estimate posterior probabilities [3]. Briggs et al. proposed a Bayesian estimation of the transition probability matrix for models with multibranch nodes [4]. In their paper flat Dirichlet prior distributions were assumed with high level of uncertainty. In their approach these flat prior probabilities from each state to the other states were combined with the clinical trial data to obtain the posterior probabilities of the transition probability matrix with Markov chain Monte Carlo (MCMC) simulation using WinBUGS [5].

The method has several advantages: (1) it solves the problem of having zero values in the transition probability matrix derived from the clinical trial, (2) assuming a high level of uncertainty of the prior probabilities ensures that the clinical trial data influences most of the posterior probabilities, (3) estimation and probabilistic sensitivity analysis can be performed in one step if the Markov modelling process is also performed within the same framework, and (4) MCMC methods allow samples to be drawn from the joint posterior density, fully considering parameter uncertainty and the correlations between parameters.

The major limitation of the method applied by Briggs et al. is that the transition probabilities that did not occur in the clinical trials are influenced only by the prior probabilities, and in this case, applying vague priors might result in inappropriate estimates. A solution for the problem may be the use of informative priors based on expert opinions. Unfortunately, eliciting a Dirichlet prior distribution is not straightforward [6]. A key challenge is satisfying all the constraints of mathematical coherence. For example, the probabilities of each category must sum to one. Another challenge is that clinical experts cannot easily and directly estimate the probabilities of multiple outcomes, as “human limitations of memory and information processing capacity often lead to subjective probabilities that are poorly calibrated or internally inconsistent, even when assessed by experts”, so the primary questions must be divided into simple questions that are easy to understand and answer [7].

Recently, a freeware application was published by Elfadaly and Garthwaite to aid in eliciting Dirichlet and Gaussian copula prior distributions [8]. The proposed method elicits hyperparameters of the Dirichlet distribution from those of its marginal beta distributions through forms of reconciliation that use least-squares techniques.

Using both of the two abovementioned methods, we estimated the transition probability matrix of the patients with predominantly negative symptoms of schizophrenia in a cost-effectiveness analysis comparing the effect of cariprazine to that of risperidone. We illustrated case-specific differences between the clinical expert elicited priors and those of more common approaches (i.e., applying non-informative priors or assuming unobserved transitions have zero probability).

## Methods

### The context of the case study is as follows

**Fig. 1:**
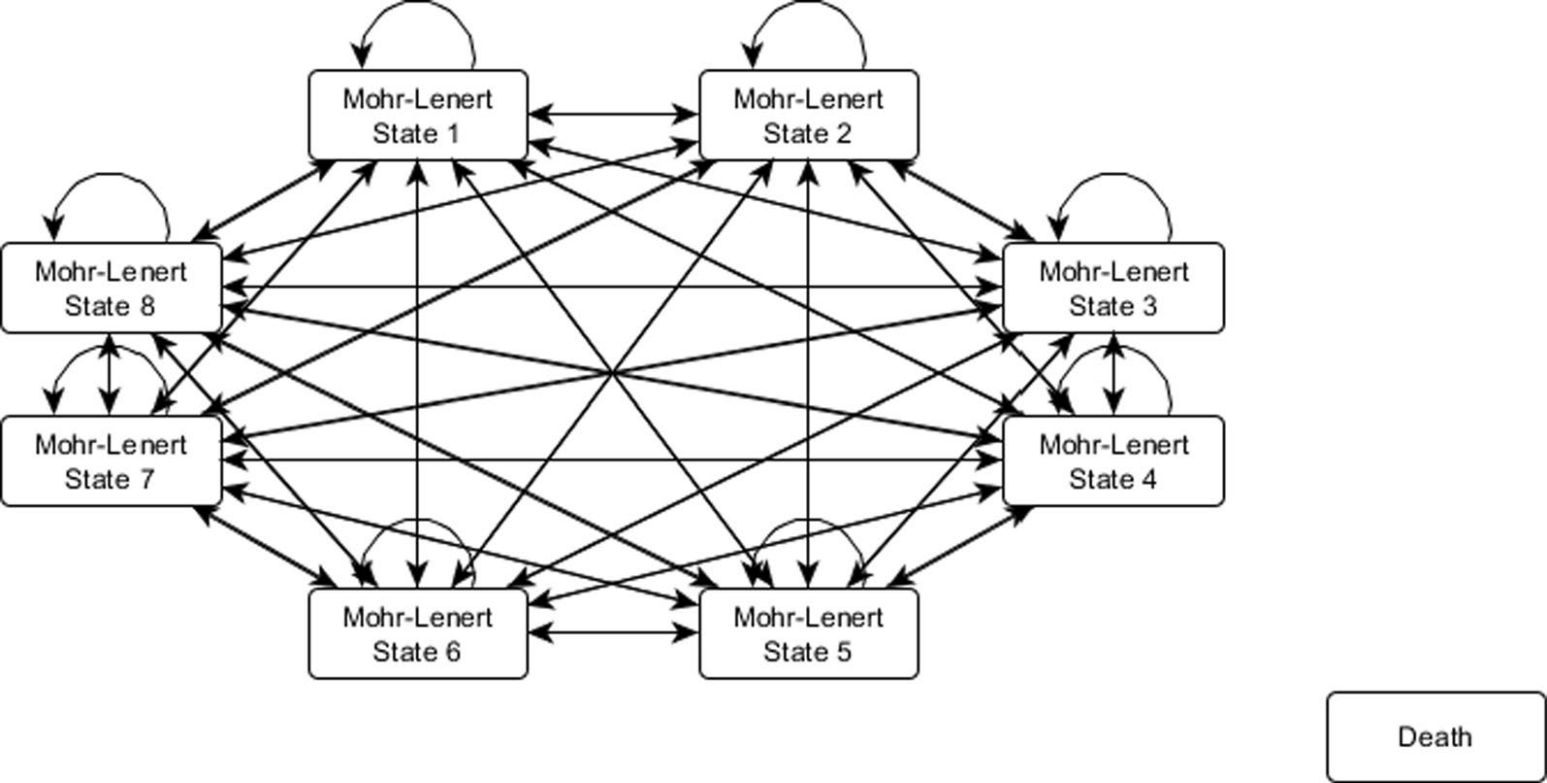
Cost-effectiveness model structure. [9] Source: Németh et al. 2017

The cost-effectiveness model has been described elsewhere (Fig. 1) [9]. Briefly, a Markov cohort model was built in Microsoft Excel with eight health states for schizophrenia (hereinafter referred to as the Mohr-Lenert health states) defined by Mohr et al. in 2004 and a death state [10]. The definition of the health states can also be found in a concise format in Table 2 of the paper presenting the cost-effectiveness model [9]. As the pivotal clinical trial in which the model was based on provided no data on mortality, as there were not any participants who died during the study period, the age- and sex-specific mortality rates of the general population were used in the model, and no difference in the mortality between the two treatment groups was assumed [11]. Considering the pharmacokinetic properties of cariprazine [12], the modelled time period was split into two periods: an initial 6-week time period with weekly cycles and 12-week long cycles occurring thereafter. Because the full clinical effects of cariprazine are expected to occur after the first 6 weeks, different transition probabilities were necessary for the first 6 weeks and for the subsequent model time period. Because the aim of this paper is to present a method of estimating the transition probability matrix for Markov models based on both expert opinion and clinical trial data, we used only the first 6-week period as an illustration of the method. The same method was applied for the subsequent period but with different observed data and prior probabilities.

Data to estimate the weekly transition probabilities for the first 6 weeks of the modelled time period for both the cariprazine and the risperidone arms were available from the first 4-week period of the Németh et al. clinical trial [11]. In the original publication, the model was used to estimate the utility of the cariprazine treatment compared to the risperidone treatment.

### Prior elicitation

The prior probabilities in the transition matrix were elicited with the involvement of three leading clinical experts from Hungary who are actively involved in treating patients with schizophrenia and have deep insight into the typical courses of the disease (IB, BM, JR), and who were involved in the project. An additional criterion at the selection was to have considerable research experience. The prior probabilities were elicited using Prior Elicitation Graphical Software (PEGS) [8]. The feasibility of using the application, and the process of the elicitation was pilot tested without the involvement of the experts. The definitions of the Mohr-Lenert health states were explained to the experts. Besides this information given verbally, and the technical description of the exercise there were not any additional information given to the experts regarding the transitional probabilities between the studied health states. Then, the experts were asked individually face-to face to give their opinion about the probabilities of patients with predominantly negative symptoms of schizophrenia moving from a given Mohr-Lenert health state to another state conditional on the comparator treatment (i.e., risperidone). The experts were aware of main results of the clinical trial regarding the efficacy of cariprazine, but they were unaware of the trial results about the transition probabilities between different Mohr-Lenert health states. At each step we verified whether the experts understood what they had to assess (i.e., the descriptions of the patients in a certain state, which was used as the current state, and that they had to estimate the proportion of patients moving from this state to another state). In the assessment process, all experts were asked to assess the marginal medians and quartiles of the probability of each transition. Because directly estimating these statistics is difficult for clinical experts, we elicited this information by asking the following questions. For the median, the question was phrased as follows: “Considering patients in state A, what is the most likely proportion of patients moving to state B within one week during the first six weeks of treatment? Consider it equally likely that the true proportion is above this value or below this value. For example, suppose you assess this value as 0.4, you should think it is equally likely that the true proportion will be above 0.4 as it will be below 0.4.” For the lower quartiles, the question was phrased as follows: “If you were told that the true proportion was smaller, what is the value that you think is still reasonable? Estimate a proportion for which it holds that the probability that the true proportion is below equals the probability that the true proportion is between it and 0.4 (the median)”. Similar questions were asked to assess the upper quartiles.

In the next step, the experts were told that “the probabilities of the different patient movements from a given health state must add to one, and the assessments must also meet certain other requirements to be internally consistent. The application gives you three options for reconciling your assessments to meet these requirements. Select the one which best represents your opinion.” Once a clinical expert made a choice about these options, he or she was confronted with the result. Then, he or she still had an opportunity to change any quartiles. After the modifications, the application again calculated the coherent marginal quartiles for the Dirichlet distribution and presented them together with the expert’s revised values. Finally, when the expert thought that the proposition was in line with his or her view, the application presented the estimated transition probabilities with their variance and the hyperparameters of the Dirichlet distribution.

As the same prior was later used for modelling patients’ paths in the cariprazine arm, the difference in treatment efficacy originated only from the trial data. The estimated transition probabilities by the three experts were averaged and scaled to one person in each source state, ensuring high uncertainty of the prior probabilities and thereby allowing a large influence of the clinical trial data.

### Estimation of the transition probability matrix

The transition probability matrix was finally estimated by WinBUGS based on the priors and the clinical evidence from the trial with 1000 burn-in samples and 50,000 estimation samples; see the code in (Additional file 1). Two chains were run, and convergence was assessed by visual inspection of the trace plots and by tracking the Brooks-Gelman-Rubin diagnostics.

We generated the treatment-specific transition probability matrices for the Markov model in three different ways: (1) based only on the observed clinical trial data (transition probabilities not observed within the trial were set to 0); (2) based on Bayesian estimation where prior transition probabilities came from experts’ opinions (as described previously); and (3) based on Bayesian estimation with vague prior transition probabilities (flat Dirichlet prior distributions). Furthermore, we compared the transition probability matrices and the incremental quality-adjusted life years (QALYs) across the three approaches.

## Results

**Fig. 2:**
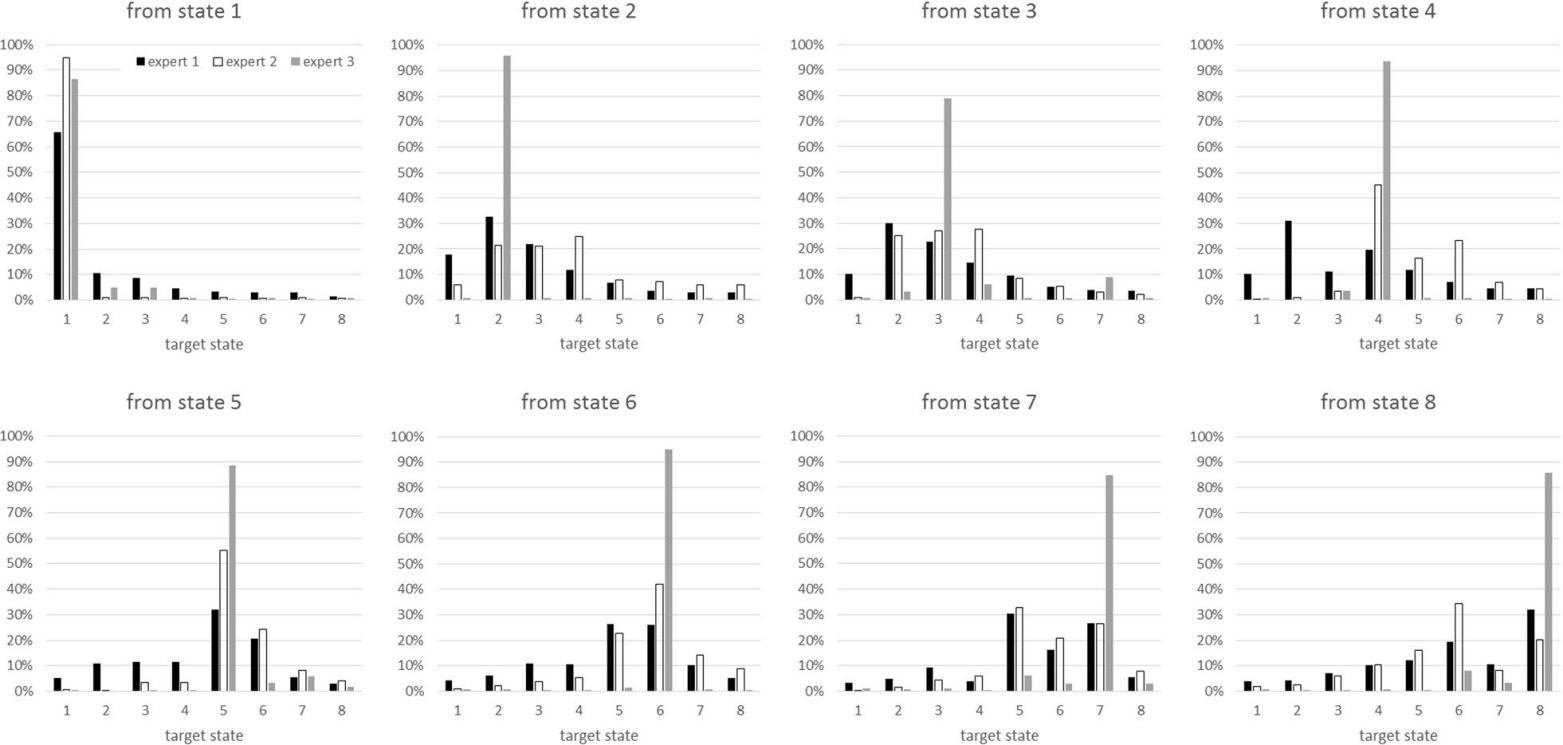
Weekly transition probability estimates determined by the experts

The experts found determining the priors mentally exhausting, as the phrasing of the questions were the same for each health state. It took approximately one hour for each expert to complete the two transition probability matrices (for the first 6 weeks and thereafter). The experts found the feedback loops in the application to be very helpful, as reviewing the results of their estimations helped them correct their initial judgements when they felt it was necessary. The estimates of experts 1 and 2 showed considerable consistency (Fig. 2), whereas expert 3 consistently estimated higher probabilities for the patients staying in the state where they were than did the other two experts. Table 1 shows the mean values of the estimates of the three experts.

**Table 1:** Weekly transition probability estimates (%) by the experts (mean values)

From [row] / to [col]	1	2	3	4	5	6	7	8
1	82.39	5.42	4.81	2.00	1.56	1.40	1.54	0.88
2	8.12	49.90	14.46	12.49	5.05	3.73	3.17	3.07
3	3.96	19.54	42.86	16.14	6.29	3.78	5.27	2.16
4	3.65	10.65	6.07	52.76	9.59	10.34	3.90	3.05
5	2.03	3.73	5.09	5.03	58.51	16.05	6.61	2.96
6	1.99	3.00	5.03	5.48	16.97	54.31	8.39	4.83
7	1.50	2.38	4.87	3.48	23.14	13.28	45.90	5.46
8	2.26	2.46	4.49	7.06	9.63	20.61	7.43	46.06

This data by expert have already been published in the appendix of our paper presenting the cost effectiveness study [9]. The probabilities add up to 100% by row. The minor differences in the second digit after the comma are because of rounding

In Table 2, the observed relative frequencies of the weekly transitions in the first four weeks of the pivotal clinical trial are shown for both treatment arms [11]. There were quite a few transitions that were not observed, and there were no patients in states 7 and 8 (the two most severe Mohr-Lenert health states) in the first four weeks of the trial. In contrast to this observation, the experts generally believed that any of the possible transitions could happen, but some of these transitions were rather unlikely according to them.

**Table 2:** Observed weekly transition relative frequencies (%) between the eight health states from the first four weeks of the RGH-188-005 clinical trial

From [row] / to [col]	1	2	3	4	5	6	7	8	n
Risperidone									
1	100.00	0.00	0.00	0.00	0.00	0.00	0.00	0.00	2
2	3.31	80.99	0.00	9.09	5.79	0.83	0.00	0.00	121
3	0.00	0.00	100.00	0.00	0.00	0.00	0.00	0.00	2
4	0.00	22.10	0.55	74.31	0.83	2.21	0.00	0.00	362
5	0.00	16.07	0.00	3.57	75.00	5.36	0.00	0.00	56
6	0.00	2.87	0.00	8.91	12.07	76.15	0.00	0.00	348
7	0.00	0.00	0.00	0.00	0.00	0.00	0.00	0.00	0
8	0.00	0.00	0.00	0.00	0.00	0.00	0.00	0.00	0
Cariprazine									
1	100.00	0.00	0.00	0.00	0.00	0.00	0.00	0.00	3
2	6.03	87.07	0.00	5.17	1.72	0.00	0.00	0.00	116
3	0.00	100.00	0.00	0.00	0.00	0.00	0.00	0.00	1
4	0.00	19.89	0.27	75.33	0.53	3.98	0.00	0.00	377
5	0.00	22.92	0.00	0.00	70.83	6.25	0.00	0.00	48
6	0.00	3.51	0.00	11.99	10.53	73.98	0.00	0.00	342
7	0.00	0.00	0.00	0.00	0.00	0.00	0.00	0.00	0
8	0.00	0.00	0.00	0.00	0.00	0.00	0.00	0.00	0

*n* number of observations

Source: Appendix of Németh et al. 2017 [9]

The average estimates of the experts showed reasonable consistency with the trial data, with some attenuation of the extreme observed values in the trial (Tables 1 and 2). For example, both patients who were in the risperidone arm who were in state 3 at the beginning of the trial stayed in state 3, resulting in a 100% transition probability, whereas the mean of the expert estimates was 42.9%. Note that if one of the two patients had moved to a different state, the trial transition probability estimate would have dropped to 50%. Similarly, the estimates for the transitions from state 1 to state 1 were 100% in both treatment arms based on the trial data, while the estimate was 82.39% by the experts. The experts did not exclude the possibility of any particular transition; thus, there were no zero estimates in their estimated transition probability matrix.

The estimates of the two chains converged on the trace plot, and the Brooks-Gelman-Rubin diagnostics did not show evidence for non-convergence either.

As expected, the posterior values of the transition probability matrix were very similar to the trial data relative frequencies in cells where there were a reasonably large number of events (states 2, 4, and 6) (Table 3). For example, in the clinical trial, the proportion of patients who stayed in state 2 was 80.99% in the risperidone arm, whereas the estimated posterior probabilities (expressed in %) were 80.72 and 80.43%, depending on the choice of the prior probability. In the case of the mildest disease state (state 1), the pattern of the posterior estimates clearly followed the pattern of the clinical trial data estimates when the informative prior was used. The posterior probability of staying in the mildest state, however, was much lower when the uninformative (flat) prior was used. This result was expected, as the experts’ opinions were in line with the observations in the clinical trial, and only 2 and 3 cases occurred in the risperidone arm and in the cariprazine arm, respectively, in the initial 4 weeks. This behaviour also occurred for the patient movements from state 3 in the risperidone arm. As the prior probability and the relative frequency from the trial had the same level of uncertainty (both were based on one case) in the cariprazine arm for state 3, the posterior probability distribution reflects equal influence of the prior probabilities and clinical trial data. As the same prior was used for both treatment arms, and there were no observations in states 7 and 8 in the initial 4 weeks of the trial, the posterior distributions of the transition probabilities from these health states were essentially the same in the two arms.

**Table 3:** Posterior estimates of the weekly transition probabilities (%) estimated with the use of uninformative (normal text) and informative (bold text) prior probabilities and clinical trial data

From [row] / to [col]	1	2	3	4	5	6	7	8
Risperidone								
1	**94.11**	**1.80**	**1.60**	**0.68**	**0.55**	**0.47**	**0.51**	**0.28**
	70.98	4.15	4.16	4.10	4.16	4.14	4.18	4.13
2	**3.35**	**80.72**	**0.12**	**9.13**	**5.78**	**0.85**	**0.03**	**0.03**
	3.39	80.43	0.10	9.13	5.83	0.93	0.11	0.10
3	**1.33**	**6.60**	**80.97**	**5.23**	**2.09**	**1.31**	**1.76**	**0.71**
	4.13	4.16	70.92	4.18	4.10	4.19	4.20	4.12
4	**0.01**	**22.07**	**0.57**	**74.24**	**0.85**	**2.23**	**0.01**	**0.01**
	0.03	22.07	0.59	74.13	0.86	2.24	0.03	0.03
5	**0.03**	**15.83**	**0.09**	**3.61**	**74.71**	**5.56**	**0.11**	**0.05**
	0.21	16.01	2.14	3.74	73.89	5.49	0.22	0.22
6	**0.01**	**2.88**	**0.01**	**8.90**	**12.08**	**76.09**	**0.02**	**0.01**
	0.04	2.89	0.04	8.92	12.07	75.97	0.04	0.04
7	**1.49**	**2.37**	**4.98**	**3.46**	**23.01**	**13.25**	**45.98**	**5.46**
	12.41	12.51	12.51	12.55	12.51	12.71	12.45	12.34
8	**2.22**	**2.51**	**4.51**	**7.04**	**9.62**	**20.56**	**7.41**	**46.15**
	12.44	12.44	12.49	12.46	12.64	12.52	12.42	12.59
Cariprazine								
1	**95.55**	**1.40**	**1.23**	**0.51**	**0.39**	**0.34**	**0.36**	**0.22**
	77.95	3.15	3.13	3.12	3.18	3.20	3.11	3.16
2	**6.05**	**86.77**	**0.12**	**5.23**	**1.74**	**0.03**	**0.03**	**0.03**
	6.09	86.42	0.11	5.24	1.83	0.11	0.11	0.11
3	**1.98**	**59.77**	**21.44**	**8.02**	**3.08**	**1.92**	**2.69**	**1.12**
	6.30	56.3	6.29	6.26	6.26	6.09	6.29	6.27
4	**0.01**	**19.86**	**0.28**	**75.28**	**0.55**	**3.99**	**0.01**	**0.01**
	0.03	19.87	0.30	75.16	0.56	4.01	0.03	0.03
5	**0.04**	**22.52**	**0.11**	**0.10**	**70.55**	**6.48**	**0.13**	**0.06**
	0.25	22.69	0.25	0.25	69.65	6.38	0.26	0.25
6	**0.01**	**3.51**	**0.02**	**11.97**	**10.55**	**73.92**	**0.02**	**0.01**
	0.04	3.59	0.04	12.00	10.54	73.78	0.04	0.04
7	**1.58**	**2.45**	**4.83**	**3.50**	**23.12**	**13.31**	**45.80**	**5.41**
	12.53	12.56	12.47	12.44	12.42	12.49	12.45	12.64
8	**2.37**	**2.48**	**4.47**	**7.18**	**9.60**	**20.59**	**7.42**	**45.90**
	12.42	12.31	12.67	12.52	12.39	12.56	12.56	12.56

Source: partially from the appendix of Németh et al. 2017 [9]

When the informative priors were used, these distributions reflected the opinions of the experts and were not at all uniform, avoiding the bias that would have resulted from the use of vague priors that assumed that the probability of each transition was the same from these health states (i.e., 1/8).

Table 4 shows the results regarding the health benefits with the use of the three different transition probability matrices. The estimated difference in QALYs was small but not negligible over a 54-week-long time period. Compared to the model that incorporated expert opinions, the model including only the clinical trial data estimated 2.8% higher, while the model using vague priors estimated 4% smaller QALY differences between the treatment arms. Nevertheless, these differences were very small compared to the precision of the QALY difference estimate, as the standard deviation of it was 0.014 after 1000 model runs in the probability sensitivity analysis in the cost-effectiveness analysis.

**Table 4:** Health benefit results of cost-effectiveness modelling with different transition probability matrices

	Source of the transition probability matrix		
	Only the clinical trial	Expert opinion + clinical trial	Vague prior + clinical trial
QALY cariprazine	0.7562	0.7540	0.7512
QALY risperidone	0.7302	0.7287	0.7269
QALY difference	0.0260	0.0253	0.0243

## Discussion

Substituting zeros with plausible and valid values in a transition probability matrix of a state transition model is challenging. In a Bayesian analysis, we combined clinical trial data with informative Dirichlet prior probabilities of transitions between health states defined by Mohr et al. in patients with predominantly negative symptoms of schizophrenia [10]. The elicitation of Dirichlet prior probabilities proved to be feasible and reliable with the application developed by Elfadaly and Garthwaite, as the average values of the experts’ estimates showed considerable consistency with the observed relative frequencies from the clinical trial for transitions that were observed in the trial. The opinions of the different experts could be pooled by linear combinations or by the supra Bayesian procedure [13]. The simple averaging method, which we used, is a widely used and recommended method, especially with small sample sizes [14].

A strength of our approach was that the evidence about treatment efficacy was derived entirely from the clinical trial, as the same prior distribution was used on the two arms. The use of informative priors for treatment efficacy might be justifiable in some cases, but we think that extracting efficacy estimates from clinical trials or effectiveness estimates from well-designed observational studies provides more valid results [15].

Different methods of estimating the transition probability matrix may lead to considerably different results. In our case, the size of the difference between the three results in the incremental QALY was not large. The size of this difference, however, depends on the specifics of the actual model. When the forecasting of the model includes significantly long periods of time in health states or transitions that were not observed or when there are large differences in the quality of life or costs of health states, the size of the difference in the results is likely to be larger. In our case, for example, when the time period of the model was extended to 258 weeks, the 4% difference in the incremental health gain between the approaches using vague and informative priors increased to approximately 6% (QALY difference of 0.0825 and 0.0875, respectively). The differences in the QALY estimates by estimation method were in line with our expectations, as the clinical experts estimated that transitions to the more severe states were less frequent than assumed with the use of the non-informative priors. Thus, we expected larger QALY estimates when informative priors were used.

Although several guidelines have been published about how to perform health economic modelling, the estimation of the transition probability matrix is a neglected area of study, as there are no available guidelines regarding this topic [16]. The Bayesian approach provides more plausible and valid results than simply assuming zero probabilities for the unobserved transitions, when these unobserved transitions still occur in clinical practice. Our case study showed that using vague priors different from the experts’ prior beliefs resulted in different model results. When the selection of the priors largely influences the model results (e.g., when the clinical trial data have large uncertainty), sensitivity analyses with plausible ranges of the priors can help determine the robustness of the results.

## Conclusions

In summary, the proposed method by Briggs et al. provides a conceptual framework and practical solution to estimate transition probability matrices when some of the possible transitions did not occur in the empirical studies [4]. Using informative priors rather than vague priors when the Bayesian approach is applied could be an option when the transition probabilities without empirical estimates have a significant impact on the model results.. The software application developed by Elfadaly and Garthwaite is a good practical aid to elicit Dirichlet and Gaussian copula priors by expert interviews [8].

## Supplementary information

**Additional file 1.** The WinBUGS code of the analysis.

## Data Availability

The supplementary material (WinBUGS program code) used to support the findings of this study are included within (Additional file 1).

## Abbreviations

MCMC: Markov chain Monte Carlo
PEGS: Prior Elicitation Graphical Software
QALY: Quality adjusted life years

## References

[1] Marsh K, Phillips CJ, Fordham R, Bertranou E, Hale J (2012). Estimating cost-effectiveness in public health: a summary of modelling and valuation methods. Health Econ Rev.

[2] Sonnenberg FA, Beck JR (1993). Markov models in medical decision making: a practical guide. Med Decis Making.

[3] O’Hagan A, Stevens JW (2002). Bayesian methods for design and analysis of cost-effectiveness trials in the evaluation of health care technologies. Stat Methods Med Res.

[4] Briggs AH, Ades AE, Price MJ (2003). Probabilistic sensitivity analysis for decision trees with multiple branches: use of the Dirichlet distribution in a Bayesian framework. Med Decis Making.

[5] Lunn D, Jackson C, Best N, Spiegelhalter D (2012). The BUGS book: a practical introduction to Bayesian analysis.

[6] Zapata-Vázquez RE, O'Hagan A, Soares BL (2014). Eliciting expert judgements about a set of proportions. J Appl Stat.

[7] Fox CR, Clemen RT (2005). Subjective probability assessment in decision analysis: partition dependence and bias toward the ignorance prior. Manage Sci.

[8] Elfadaly FG, Garthwaite PH (2017). Eliciting Dirichlet and Gaussian copula prior distributions for multinomial models. Stat Comput.

[9] Németh B, Molnár A, Akehurst R, Horváth M, Kóczián K, Németh G, Götze Á, Vokó Z (2017). Quality-adjusted life year difference in patients with predominant negative symptoms of schizophrenia treated with cariprazine and risperidone. J Comp Eff Res.

[10] Mohr PE, Cheng CM, Claxton K, Conley RR, Feldman JJ, Hargreaves WA, Lehman AF, Lenert LA, Mahmoud R, Marder SR, Neumann PJ (2004). The heterogeneity of schizophrenia in disease states. Schizophr Res.

[11] Németh G, Laszlovszky I, Czobor P, Szalai E, Szatmári B, Harsányi J, Barabássy Á, Debelle M, Durgam S, Bitter I, Marder S (2017). Cariprazine versus risperidone monotherapy for treatment of predominant negative symptoms in patients with schizophrenia: a randomised, double-blind, controlled trial. Lancet.

[12] Citrome L (2013). Cariprazine: chemistry, pharmacodynamics, pharmacokinetics, and metabolism, clinical efficacy, safety, and tolerability. Expert Opin Drug Metab Toxicol.

[13] Jacobs RA (1995). Methods for combining experts' probability assessments. Neural Comput.

[14] de Menezes LMW, Bunn D, Taylor JW (2000). Review of guidelines for the use of combined forecasts. Eur Jour Operational Res.

[15] Cooper NJ, Sutton AJ, Abrams KR (2002). Decision analytical economic modelling within a Bayesian framework: application to prophylactic antibiotics use for caesarean section. Stat Methods Med Res.

[16] Olariu E, Cadwell KK, Hancock E, Trueman D, Chevrou-Severac H (2017). Current recommendations on the estimation of transition probabilities in Markov cohort models for use in health care decision-making: a targeted literature review. Clinicoecon Outcomes Res.

