# Supplementary material for: Using informative prior based on expert opinion in Bayesian estimation of the transition probability matrix in Markov modelling—an example from the cost-effectiveness analysis of the treatment of patients with predominantly negative symptoms of schizophrenia with cariprazine
Source: Cost Eff Resour Alloc. 2020 Aug 27;18:28. doi: 10.1186/s12962-020-00224-w (PMC7457290; doi:10.1186/s12962-020-00224-w)
Supplement: Supplementary file 1 — Additional file 1. The WinBUGS code of the analysis. [file 12962_2020_224_MOESM1_ESM.docx]

**The WinBUGS code of the analysis**

model{

#Multinomial distribution for events, for the i=8 non-absorbing states

for(i in 1:8){

r.car1w[i,1:8] ~ dmulti(p.car1w[i,1:8], n.car1w[i])

r.ris1w[i,1:8] ~ dmulti(p.ris1w[i,1:8], n.ris1w[i])

}

#Dirichlet prior distributions for the transition probabilities

for(i in 1:8){

p.car1w[i,1:8] ~ ddirch(prior.ris1w[i,1:8]) #same prior on both arms

p.ris1w[i,1:8] ~ ddirch(prior.ris1w[i,1:8])

}

}

#Data

list(r.car1w=structure(.Data=c(3,0,0,0,0,0,0,0, 7,101,0,6,2,0,0,0, 0,1,0,0,0,0,0,0, 0,75,1,284,2,15,0,0, 0,11,0,0,34,3,0,0, 0,12,0,41,36,253,0,0, 0,0,0,0,0,0,0,0, 0,0,0,0,0,0,0,0),.Dim=c(8,8)),

n.car1w=c(3,116,1,377,48,342,0,0),

r.ris1w=structure(.Data=c(2,0,0,0,0,0,0,0, 4,98,0,11,7,1,0,0, 0,0,2,0,0,0,0,0, 0,80,2,269,3,8,0,0, 0,9,0,2,42,3,0,0, 0,10,0,31,42,265,0,0, 0,0,0,0,0,0,0,0, 0,0,0,0,0,0,0,0),.Dim=c(8,8)),

n.ris1w=c(2,121,2,362,56,348,0,0),

prior.ris1w=structure(.Data=c(0.824,0.054,0.048,0.02,0.016,0.014,0.015,0.009, 0.081,0.499,0.145,0.125,0.05,0.037,0.032,0.031, 0.04,0.195,0.429,0.161,0.063,0.038,0.053,0.022, 0.037,0.107,0.061,0.528,0.096,0.103,0.039,0.031, 0.02,0.037,0.051,0.05,0.585,0.160,0.066,0.03, 0.02,0.03,0.05,0.055,0.17,0.543,0.084,0.048, 0.015,0.024,0.049,0.035,0.231,0.133,0.459,0.055, 0.023,0.025,0.045,0.071,0.096,0.206,0.074,0.461),.Dim=c(8,8))

)
